# A High-Risk Patient With COVID-19 Vaccine Hesitancy Successfully Treated With Monoclonal Antibodies Through Two Major Surges

**DOI:** 10.7759/cureus.22721

**Published:** 2022-02-28

**Authors:** Hitesh Gurjar, Haider Ghazanfar, Asim Haider, Nolberto Hernandez, Abhilasha Jyala, Sridhar Chilimuri

**Affiliations:** 1 Internal Medicine, BronxCare Health System, Bronx, USA; 2 Internal Medicine/Gastroenterology, BronxCare Health System, Bronx, USA

**Keywords:** covid-19, vaccine hesitancy, sotrovimab, bamlanivimab, monoclonal antibodies

## Abstract

Vaccine hesitancy remains a significant challenge in managing the current pandemic despite highly effective vaccines in the United States. Monoclonal antibodies (mAb) are an essential addition to coronavirus disease 2019 (COVID-19) treatment, along with oral antiviral agents (OAA), for non-hospitalized patients having risk factors for progression to severe COVID-19, especially in unvaccinated people. We present a case of a 74-year-old unvaccinated Hispanic woman with a history of diabetes mellitus, hypertension, coronary artery disease, obesity, and asthma who survived two episodes of severe acute respiratory syndrome coronavirus 2 (SARS‑CoV‑2) infections in January 2021 and December 2021 with exclusive use of mAb. Our case highlights the importance of using mAbs for treating high-risk patients with SARS-CoV-2 infection, especially in patients with vaccine hesitancy.

## Introduction

Despite the wide availability of highly effective coronavirus disease 2019 (COVID-19) vaccines, the United States continues to see major surges of severe acute respiratory syndrome coronavirus 2 (SARS‑CoV‑2) infections due to vaccine hesitancy. The CDC reports that unvaccinated patients account for a vast majority of SARS‑CoV‑2 deaths. Monoclonal antibodies (mAb) are an important addition to COVID-19 treatment along with oral antiviral agents (OAA) for non-hospitalized patients with risk factors for progression to severe COVID-19 [1]. 

These antibodies bind to the spike protein of SARS-CoV-2 and prevent its entry and further disease worsening [2]. They are currently approved for use in adult and pediatric patients, who are ≥12 years old and weigh at least 40 kg, and tested positive for SARS-CoV-2 with risk factors for progression of the disease to severe COVID-19. These are not authorized for hospitalized patients, patients requiring oxygen, or increasing oxygen requirements for those on long-term oxygen therapy. We present a case of a 74-year-old unvaccinated Hispanic woman who survived two episodes of COVID-19 infection with mAbs, despite multiple major risk factors, demonstrating the value of mAb. Resistance to mAb has emerged, with only sotrovimab and bebtelovimab remaining effective against the omicron variant of SARS‑CoV‑2.

## Case presentation

Our patient is a 74-year-old Hispanic woman who first presented in January 2021 with body aches, headache, loss of smell, change in taste, dry cough, and fever of five-day duration. Her past medical history was significant for diabetes mellitus, hypertension, coronary artery disease, obesity, and asthma. Past surgical history was significant for coronary artery bypass, hysterectomy, and cystocele repair. Her family history was remarkable for hypertension and diabetes. She had no known drug allergies. She denied smoking, alcohol abuse, or use of recreational drugs. She had not received any COVID-19 vaccine. Her nasopharyngeal polymerase chain reaction (PCR) swab was positive for SARS-CoV-2. On physical examination, her temperature was 98.4 Fahrenheit (F), respiratory rate was 16 breaths per minute, a saturation of 97% on room air, pulse rate of 56 beats per minute, and blood pressure of 113/61 mmHg. Her body mass index (BMI) was 35.6 kg/m^2^. She appeared lethargic on examination and had bilateral crackles on lung auscultation. Cardiac examination revealed normal S1 and S2 heart sounds. Abdominal and neurological examination was unremarkable. She was offered mAb infusion, given her several risk factors for disease progression. Her initial laboratory findings are presented in Table 1. Her SARS-CoV-2 nucleocapsid antibodies were non-reactive as detected by Elecsys Anti-SARS-CoV-2 test (Roche Diagnostics International Ltd., Rotkreuz, Switzerland). Since the dominant strain was Alpha variant she agreed and received bamlanivimab infusion. She did not experience any side effects from the mAb infusion. She reported that fever, body aches, headaches, loss of smell, and change in taste resolved after 24 hours of infusion. Her cough resolved on the third day after the mAb infusion.

One year later, she presented again with a dry cough, headache, fever, loss of smell, and change in taste for three days. She denied getting sick or hospitalized since the previous episode. She denied receiving any vaccination for COVID-19 in the interim. Her nasopharyngeal PCR swab was positive for SARS‑CoV‑2. At presentation, her temperature was 98.4 F, respiratory rate was 15 breaths per minute, a saturation of 98% on room air, pulse rate of 72 beats per minute, and blood pressure of 144/91 mmHg. She had incessant coughing and appeared lethargic on general examination. She had bilateral vesicular breathing on lung auscultation. Cardiac examination revealed normal S1 and S2 heart sounds. Abdominal and neurological examination was unremarkable. Since she had multiple risk factors and worsening symptoms, she was offered mAb infusion. Her laboratory findings are summarized in Table 1.

**Table 1:** Summary of Laboratory Findings During Both Clinical Encounters SARS-CoV-2: Severe acute respiratory syndrome coronavirus 2

Laboratory Investigation	First Encounter	Second Encounter	Reference Range
White Blood Cell Count (K/ul)	4.0	6.1	4.8 - 10.8
Absolute Lymphocyte Count (K/ul)	2.5	2.4	1.0 - 4.8
Hemoglobin (g/dl)	11.8	11.5	12.0 - 16.0
Hematocrit (%)	36.1	35.4	42.0 - 51.0
Platelet count (K/ul)	177	205	150 - 400
Serum Sodium (mEq/l)	133	136	135 - 145
Serum Potassium (mEq/l)	4.2	4.1	3.5 - 5.0
Serum bicarbonate (mEq/l)	31	27	24 - 30
Serum Chloride (mEq/l)	96	98	98 - 108
Blood Urea Nitrogen (mEq/l)	9.0	9.0	6.0 - 20.0
Serum Creatinine (mEq/l)	1.0	1.0	0.5 - 1.5
Lactate Dehydrogenase (unit/L)	211	371	110 - 210
C-Reactive Protein (mg/L)	< 5.00	13.31	≤ 5.00
D-Dimer (ng/mL)	< 150	157	0 - 230
Ferritin (ng/mL)	101. 0	77.9	13-150
SARS-CoV-2 nucleocapsid antibodies	Non-reactive	Reactive	Non-Reactive
SARS-CoV-2 spike antibodies	Not available	22.50 U/mL	> = 0.00 U/mL

Her SARS-CoV-2 nucleocapsid antibodies detected by Elecsys Anti-SARS-CoV-2 test (Roche Diagnostics International Ltd., Rotkreuz, Switzerland) were reactive and her SARS-CoV-2 spike antibody level (detected using Elecsys Anti SARS-CoV-2 S assay {Roche Diagnostics International Ltd., Rotkreuz, Switzerland}) were 22.50 U/mL (reference range > = 0.00 U/mL). In June 2021, the Delta variant became dominant in New York, followed by the Omicron variant in December 2021, which has been reported to evade the immunity obtained from prior infection and vaccination. Sotrovimab was also reported as the only effective mAb for the Omicron variant. She received sotrovimab mAb infusion without any adverse effects. She reported symptom improvement after 24 hours of mAb infusion, with complete resolution after 48 hours of mAb infusion. She denied any adverse event or hospitalization on the 30th-day follow-up after infusion.

## Discussion

**Figure 1:**
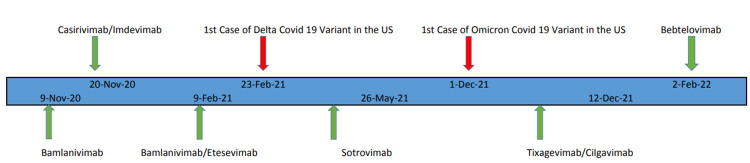
Timeline depicting Emergency Use Authorization of Monoclonal Antibodies and Emergence of SARS‑CoV‑2 Variant SARS‑CoV‑2: Severe acute respiratory syndrome coronavirus 2 COVID 19: Coronavirus disease 2019 US: United States

Monoclonal antibodies bind to the spike protein of SARS‑CoV‑2 and prevent its entry and further disease worsening [2]. Bamlanivimab (November 9, 2020), bamlanivimab with etesevimab (February 9, 2021), casirivimab and imdevimab (November 21, 2020), sotrovimab (May 26, 2021) and bebtelovimab (February 11, 2022) were approved under emergency use authorization (EUA) by U.S. Food and Drug Administration (FDA) (parentheses include the date of EUA approval respectively). The timeline of EUA approval of mAbs and the emergence of the SARS‑CoV‑2 variant is depicted in Figure 1.

Due to the emergence of resistance, the use of bamlanivimab as a single agent was revoked [3]. Subsequently, EUA has withdrawn for casirivimab and imdevimab due to lack of effectiveness against the Omicron variant. In December 2021, the Omicron variant became the predominant infection in New York. Sotrovimab is the only mAb effective against Omicron. First case of Omicron variant was detected on December 2, 2021, in New York City. Within five weeks, it became the predominant strain (isolated in 90% of samples) compared to the delta variant, which required 20 weeks to become the predominant strain [4].

As of January 22, 2022, 75.5% of the U.S. population has been vaccinated [5]. However, this figure is lower in certain regions, such as the Bronx County, where our patient resides [6]. Vaccine hesitancy appears high in certain communities in the U.S. It has been reported in 30.2 % and 41.6% of Hispanics and African American populations, respectively [7]. Likewise, the use of mAb is also reportedly very low in Hispanics and African Americans [8]. Our case illustrates the effectiveness of mAb in treating a patient with vaccine hesitancy in this specific population.

She was treated with bamlanivimab in January 2021. The protection provided by mAbs usually lasts for 90 days, after which vaccine is recommended [9]. In our case, she refused the COVID-19 vaccine but readily agreed to receive mAb (sotrovimab) in December 2021. This case highlights certain important considerations. The use of mAb is a safe and effective treatment for individuals with high-risk conditions for disease progression. Age over 65 (Odd ratio:6.01), Hispanic race (Odds Ratio: 1.35), diabetes mellitus (Odd Ratio: 3.68), coronary artery disease (Odds Ratio: 3.23), and obesity (Odds Ratio: 4.17) have been associated with increased risk of progression to severe illness [10-14].

Many mAbs have been used in a variety of different clinical conditions, and their use has been associated with short-term side effects such as hypersensitivity reactions and long-term side effects such as infections, cancers, autoimmune conditions, and organ-specific toxicities [15]. We have not seen any long-term adverse events in our own experience of nearly 100 patients [16].

Vaccine hesitancy remains a major concern in the United States. Unvaccinated patients remain the largest group of patients currently hospitalized and account for the largest group of mortalities [17]. While the reasons for vaccine hesitancy appear to be multifactorial, it should not preclude physicians from offering mAbs in patients with high-risk factors for the progression of the disease [18]. These mAbs are well tolerated and are highly effective, as demonstrated in our patient. While effective, mAbs should not be inferred as an alternative to the vaccine, as indeed, vaccinations remain the primary goal. Introduction of new long-acting mAb (tixagevimab + cilgavimab) may be used in patients as pre-exposure prophylaxis in patients with specific contraindication for vaccine or those who have anticipated inadequate response to vaccine due to immunosuppression [19]. It is possible that when ample supplies become readily available, these can be used in a wider population.

## Conclusions

Vaccine hesitancy remains a major hurdle in the management of the pandemic. However, the availability of mAb should provide effective treatment in patients with risk factors for progression, especially in the unvaccinated. While the greater goal is to increase vaccination rates, unvaccinated patients with risk factors should be offered mAb to prevent the progression of the disease. Our case highlights the importance of offering mAb to patients earlier in the disease course as it is associated with better clinical outcomes. Despite being at high risk for progression to severe COVID-19, our patient did well on mAb infusions and adverse outcomes were avoided. 
